# *LINC00857* expression predicts and mediates the response to platinum‐based chemotherapy in muscle‐invasive bladder cancer

**DOI:** 10.1002/cam4.1570

**Published:** 2018-06-01

**Authors:** Aleksandra M. Dudek, Jasmijn G. M. van Kampen, J. Alfred Witjes, Lambertus A. L. M. Kiemeney, Gerald W. Verhaegh

**Affiliations:** ^1^ Department of Urology Radboud Institute for Molecular Life Sciences Radboud university medical center Nijmegen The Netherlands; ^2^ Department for Health Evidence Radboud Institute for Health Sciences Radboud university medical center Nijmegen The Netherlands

**Keywords:** biomarker, cisplatin, *LINC00857*, long non‐coding RNAs, muscle‐invasive bladder cancer

## Abstract

Approximately 20% of patients with bladder cancer are diagnosed with muscle‐invasive disease (MIBC). The treatment involves radical cystectomy, but almost 50% of patients with MIBC eventually relapse and develop metastasis. The use of platinum‐based chemotherapy in the neoadjuvant setting or for metastatic patients has been shown to improve the overall survival in a subset of patients. Unfortunately, no biomarkers are available to select patients with MIBC who will benefit from chemotherapy or to monitor the efficacy of the treatment. Recently, long noncoding RNAs (lncRNAs) were shown to regulate a variety of processes involved in the development and progression of cancer, including bladder cancer. Moreover, several lncRNAs have been shown to play a role in chemotherapy resistance. Here, we analyzed lncRNA expression associated with response to platinum‐based chemotherapy in metastatic MIBC using data from the MiTranscriptome lncRNA expression database. Expression of the lncRNA,*LINC00857*, was found to be upregulated in tumors from patients that did not respond to platinum‐based chemotherapy. Moreover, high expression of *LINC00857* is correlated with shorter recurrence‐free and overall survival of patients with MIBC. Knockdown of *LINC00857* significantly decreased cell viability of bladder cancer cell lines through the induction of apoptosis. Furthermore, *LINC00857* knockdown sensitized UM‐UC‐3 and T24 bladder cancer cells to cisplatin, via the negative regulation of the *LMAN1* gene. Our data indicate that *LINC00857* plays an important role in the regulation of response to platinum‐based chemotherapy. *LINC00857* potentially could serve as a novel prognostic and predictive biomarker and might be a therapeutic target to overcome cisplatin resistance in patients with MIBC.

## 1 INTRODUCTION

Urinary bladder cancer ranks as the ninth most common cancer, with approximately 430 000 new cases and 165 000 deaths every year worldwide.^1^ About 20% of the patients are diagnosed with muscle‐invasive bladder cancer (MIBC), which invades through the lamina propria into the muscle layer (stage T2), perivesical tissue (stage T3), or adjacent organs (stage T4).^2^ Radical cystectomy is the gold standard treatment for MIBC. However, almost 50% of patients with MIBC relapse and develop local or distant metastasis, and the 5‐year recurrence‐free survival is approximately 74% for patients with T2 tumors, 52% for T3 tumors, and 36% for T4 tumors. The use of neoadjuvant cisplatin‐based chemotherapy (NAC) has been shown to improve the overall survival (OS) by 5%‐8%. The treatment of metastatic MIBC also involves cisplatin‐based chemotherapy. Unfortunately, roughly half of the patients that are treated with cisplatin‐based chemotherapy show a treatment response. These patients have a prolonged survival up to 14 months.^3^, ^4^

The selection of patients with MIBC who will benefit from chemotherapy represents a major challenge in clinical practice. Multiple studies have evaluated prognostic and predictive biomarkers in MIBC.^5^, ^6^ For example, somatic alterations in DNA damage response and DNA repair (DDR) genes have been associated with improved response to platinum‐based chemotherapy in advanced MIBC.^7^ Also, the prognostic and predictive value of p53 alterations has been widely investigated, but neither a prognostic value of p53 nor a benefit of methotrexate, vinblastine, doxorubicin, and cisplatin (MVAC) chemotherapy in patients with p53‐positive tumors was found.^8^ Changes in circulating tumor cells (CTCs) were shown to correlate with the clinical outcome of metastatic patients with bladder cancer after MVAC chemotherapy, as CTC‐positive patients showed significantly shorter progression‐free and overall survival.^9^ Recently, several groups have identified molecular subtypes of bladder cancer, and these were found to influence the response to NAC. The basal subtype of MIBC showed the highest benefit in OS in comparison with the luminal, luminal‐infiltrated, and claudin‐low subtypes.^10^ Until now, however, there is not enough evidence supporting clinical use of these biomarkers.^3^, ^4^ Therefore, conformation of these studies or discovery of new biomarkers for MIBC is needed to identify responders to chemotherapy, monitor the treatment, and predict patient outcome.

Long noncoding RNAs (lncRNAs) are non‐protein‐coding transcripts of at least 200 nucleotides that are expressed by RNA polymerase II activity and that undergo splicing and polyadenylation. LncRNAs outnumber the protein‐coding genes, and their expression is often lineage‐specific and in malignancies even cancer‐specific.^11^ Many lncRNAs are able to modify gene expression at different levels, depending on their cellular localization, either through interactions with chromatin modifiers and regulation of transcription in *cis* or *trans* or through the regulation of mRNA stability and protein translation.^12^ LncRNAs were found to be involved in the regulation of all different hallmarks of cancer including cell proliferation, cell viability, growth suppression, cell motility, and angiogenesis.

Several lncRNAs were shown to modify chemotherapy response in bladder cancer. For example, *UCA1* expression levels were found to be increased upon cisplatin treatment, and through activation of Wnt signaling, *UCA1* contributed to chemoresistance.^13^ *UCA1* was also shown to activate the CREB transcription factor and *miR‐196a‐5p*, the latter modulating apoptosis induced by combined gemcitabine and cisplatin (Gem/Cis) treatment.^14^ Additionally, *GAS5* 15 and *HOTAIR* 16 were found to be prognostic biomarkers. *GAS5* and *HOTAIR* expression levels were found to be deregulated in bladder cancer, and their expression was shown to be associated with tumor grade and disease‐free survival.^15^, ^16^ Moreover, modulation of *GAS5* and *HOTAIR* expression levels was found to affect proliferation of bladder cancer cells and to sensitize them to doxorubicin.^15^, ^16^

Biomarkers for the accurate selection of patients with MIBC who may benefit from platinum‐based chemotherapy are lacking. LncRNAs regulate a variety of cellular processes involved in cell survival and chemoresistance, and their expression has been shown to be deregulated in bladder cancer. Therefore, we analyzed expression levels of lncRNAs using the MiTranscriptome lncRNA expression database^11^ to identify new noncoding biomarkers that are associated with the response to platinum‐based chemotherapy in MIBC. The effect of the identified lncRNA, *LINC00857*, on cisplatin sensitivity was further characterized in vitro using loss of function studies in bladder cancer cells.

## 2 MATERIALS AND METHODS

### 2.1 Identification of lncRNAs associated with platinum‐based chemotherapy response

LncRNAs expression data for MIBC and normal urothelium were downloaded from the MiTranscriptome database (http://mitranscriptome.org).^11^ Protein‐coding gene expression data and clinical data (including response to chemotherapy) were downloaded from FIREHOSE Broad GDAC (*Broad Institute TCGA Genome Data Analysis Center (2016): Firehose 2016 01 28 run. Broad Institute of MIT and Harvard*. https://doi.org/10.7908/c11g0km9). Patients’ characteristics are listed in Table S1. Filtering of the data was performed as described previously,^17^ and lncRNAs associated with chemotherapy response were selected from the 12 037 lncRNAs (unpaired *t* test).

### 2.2 Association between gene expression and survival

The association between *LINC00857* and *LINC00857* target gene expression levels and overall survival (OS) of MIBC was analyzed using the Oncolnc portal (http://www.oncolnc.org). Recurrence‐free survival (RFS) data were downloaded from cBioPortal.^18^, ^19^ Kaplan‐Meier curves were compared using a log‐rank test (GraphPad Prism), and Cox proportional hazard regression analysis was performed.

### 2.3 Cell culture

UM‐UC‐3 (ATCC: CRL‐1749) and T24 (ATCC: HTB‐4) bladder cancer cell lines were maintained in RPMI‐1640 medium (Invitrogen) supplemented with 10% fetal calf serum (Sigma‐Aldrich, F7524) and L‐glutamine and cultured in at 37°C and 5% CO_2_. Cell lines were authenticated using the PowerPlex 21 PCR kit (Promega) by Eurofins Genomics, and all cell cultures were mycoplasma‐free.

### 2.4 siRNA‐mediated knockdown of *LINC00857* and gene expression

Silencer Select small interfering RNAs (siRNAs) directed against *LINC00857*,^20^ *LMAN1*,* PPP2R5E*, and a Silencer Select negative control No.1 siRNA were used (Ambion/Thermo Fisher Scientific; Table S2). T24 and UM‐UC‐3 bladder cancer cell lines were transfected with siRNAs at a 20 nM final concentration using Lipofectamine RNAiMAX transfection reagent (Thermo Fisher Scientific), according to the manufacturer’s instructions.

### 2.5 RNA isolation and RT‐qPCR

Total RNA was isolated using TRIzol reagent (Invitrogen), according to manufacturer’s instructions. RNA was first treated with DNase I (Invitrogen), and then random‐primed cDNA was synthesized using SuperScript II reverse transcriptase (Invitrogen). Gene expression levels were determined by SYBR Green qPCR (Roche) using a LightCycler LC480 instrument (Roche). Relative gene expression levels were calculated using the ΔΔCt method using the *HP1BP3* and *GAPDH* genes for normalization. Primer sequences are listed in Table S2.

### 2.6 Cell viability assay

Cell viability was evaluated at regular time points after transfection (48 hours) using the CellTiter‐Glo luminescence assay (Promega), according to the manufacturer’s instructions. Luminescence was measured on a Victor^3^ multilabel reader (Perkin Elmer). All experiments were performed in triplicate.

### 2.7 Colony formation assay

One day after transfection, cells were seeded in 6‐well plates at low density and cultured at standard conditions for 2 weeks. Colonies were fixed with 3% paraformaldehyde and stained with 0.01% crystal violet (Merck). The colonies were analyzed using ImageJ software. All experiments were performed in triplicate.

### 2.8 Apoptosis assay

Two days after transfection, caspase‐3/7 activity was measured using the Apo‐ONE Homogeneous Caspase‐3/7 Assay (Promega), according to the manufacturer’s instructions. The luminescence signals were measured on a Victor^3^ multilabel reader (Perkin Elmer) and normalized to the input, that is, cell numbers (assessed by CellTiter‐Glo assay). All experiments were performed in triplicate.

### 2.9 Cisplatin sensitivity assay

Two days after transfection cells were treated with increasing doses of cisplatin (Sigma). Cell viability was assessed using 3‐(4,5‐dimethylthiazol‐2‐yl)‐2,5‐diphenyl‐2H‐tetrazolium bromide (MTT) assay 24 and 48 hours after cisplatin treatment. After 4 hours of incubation, the medium containing MTT reagent was removed and DMSO (Sigma) was added. The optical density (OD) was measured at 595 nm on an iMark microplate reader (Bio‐Rad). The data were compared to nontreated cells. All experiments were performed in triplicate.

## 3 RESULTS

### 3.1 Identification of lncRNAs associated with platinum‐based chemotherapy response and MIBC progression

**Figure 1:**
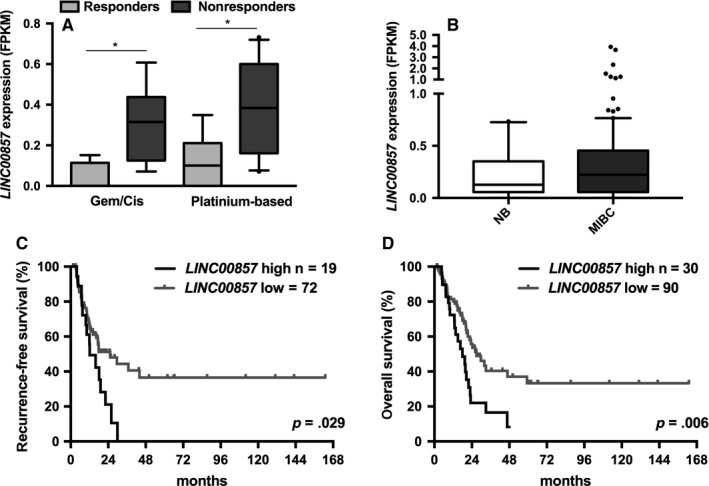
*LINC00857* expression in MIBC. Association of *LINC00857* expression with response to (A) gemcitabine/cisplatin (Gem/Cis; responders, n = 4; nonresponders, n = 7) and platinum‐based chemotherapy (responders, n = 7; nonresponders n = 10). B, *LINC00857* expression in normal urothelium (n = 16) and MIBC (n = 122). Expression data were analyzed using unpaired *t* test (*P < .05; line represent the median; the whiskers represent 10th and 90th percentile). Kaplan‐Meier analysis showing correlation between *LINC00857* expression levels with (C) recurrence‐free and (D) overall survival in MIBC MiTranscriptome cohort. The high and low *LINC00857* expression groups were determined based on the 75th and 25th percentiles. The survival curves were analyzed using the log‐rank test

To identify long noncoding RNAs that are associated with chemotherapy response, we analyzed lncRNA expression levels in patients with MIBC who received cisplatin‐based chemotherapy after cystectomy. LncRNA expression data in MIBC were derived from the MiTranscriptome dataset described by Iyer et al^11^. We identified 154 and 225 lncRNAs that are significantly associated with the response to Gem/Cis or platinum only treatment, respectively (Table S3). Previously, *LINC00857* was identified as one of the most upregulated lncRNAs in lung cancer, and high *LINC00857* expression was associated with shorter overall survival of patients with lung cancer.^20^ Therefore, we decided to further focus on *LINC00857*. *LINC00857* expression was found to be increased in MIBC in patients that did not respond to Gem/Cis (*P* = .032) nor to platinum‐based chemotherapy (*P* = .011; Figure 1A).

Although *LINC00857* was not significantly upregulated in MIBC in comparison with normal urothelium (Figure 1B), significant differences in recurrence‐free (RFS, *P* = .021; Figure S1A) and overall survival (OS, *P* = .047; Figure S1B) were observed when patients were divided into 2 subgroups based on median *LINC00857* expression. As high *LINC00857* expression was associated with chemotherapy resistance, patients with MIBC were further divided into 2 groups: patients with high (>75th percentile) and patients with low (<75th percentile) *LINC00857* expression. Patients with MIBC with high *LINC00857* expression were found to have significantly shorter RFS (*P* = .029; Figure 1C) with median 12.2 months in comparison with 25.2 months for patients with lower *LINC00857* expression. *LINC00857* expression was found also to be associated with significant decrease in median OS, decreasing from 26.9 months in the low expression group to 18 months in the high expression group (*P* = .006; Figure 1D). Moreover, high *LINC0085* expression was found to be associated with positive lymph nodes at the time of diagnosis and with more frequent progression after cystectomy (*P* = .003 and *P* = .019, resp.; Table S4). In multivariate analysis, *LINC00857* expression remained an independent predictor of OS (*P* = .025, Table S5) but not RFS (*P* = .317, Table S6).

### 3.2 *LINC00857* knockdown decreases survival of bladder cancer cells

**Figure 2:**
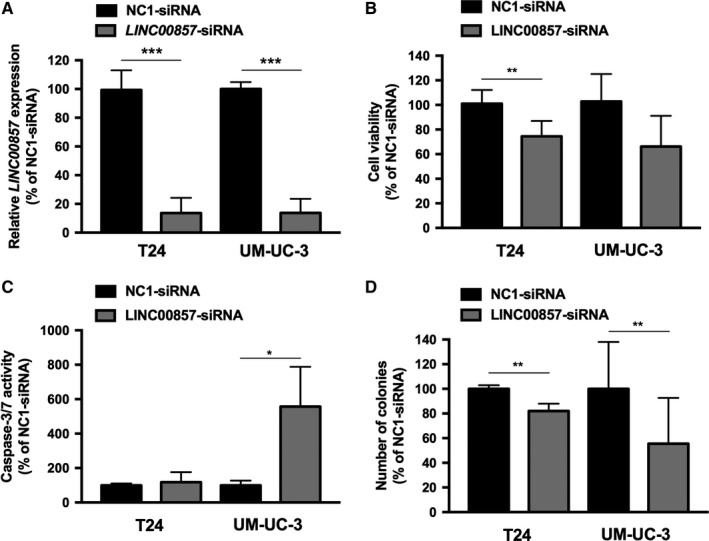
Knockdown of *LINC00857* expression in T24 and UM‐UC‐3 bladder cancer cell lines. A, The *LINC00857* expression levels 48 hours after siRNA‐mediated knockdown. The effect of *LINC00857* knockdown on (B) cell viability, (C) induction of apoptosis analyzed by caspase 3/7 activity, and (D) number of colonies formed in a colony‐forming assay (evaluated 2 weeks after transfection). All data are compared to negative control (NC1) siRNA‐transfected cells. All bars represent the mean ± SD (*t* test, *P < .05; **P < .01; ***P < .001)

To investigate the role of *LINC00857* in bladder cancer, an siRNA^20^ was used to knock down its expression in 2 muscle‐invasive bladder cancer‐derived cell lines, T24 and UM‐UC‐3 (Figure 2A). Reduced expression of *LINC00857* significantly reduced the viability of T24 cells (*P* = .003; Figure 2B), although no increase in caspase 3/7 activity was observed (*P* = .618; Figure 2C). The decrease in cell viability of UM‐UC‐3 cells (*P* = .130; Figure 2B), was accompanied by a firm induction of apoptosis, marked by high caspase 3/7 activity (*P* = .027; Figure 2C). Colony‐forming assays showed that *LINC00857* knockdown impaired growth of cancer cells marked by a reduction in the number of colonies formed by T24 and UM‐UC‐3 cells (*P* = .01 and *P* = .005, resp.; Figure 2D).

### 3.3 Knockdown of *LINC00857* sensitizes bladder cancer cells to cisplatin

**Figure 3:**
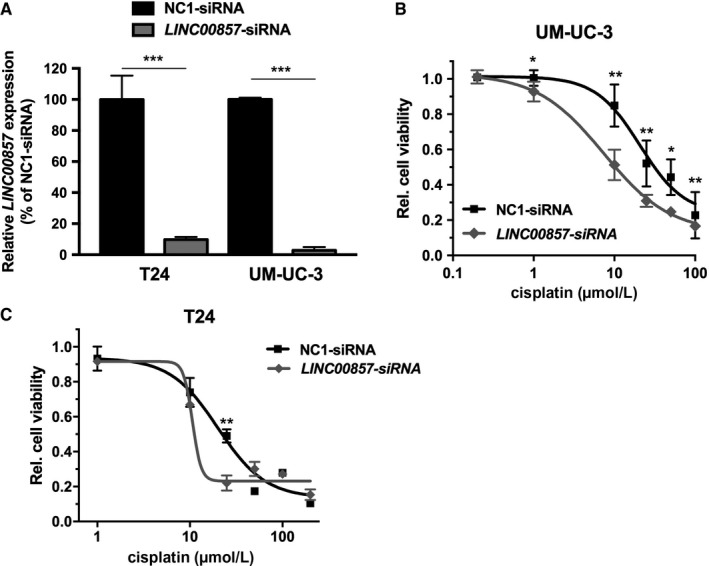
Effect of *LINC00857* on cisplatin sensitivity. A, The *LINC00857* knockdown efficiency in T24 and UM‐UC‐3 cells was determined by RT‐PCR analysis, 48 h after transfection. Gene expression levels were normalized against *GAPDH* housekeeping gene expression levels and then compared to negative control (NC1) siRNA‐transfected cells. Cisplatin sensitivity of (B) T24 and (C) UM‐UC‐3 cells 48 h after siRNA‐mediated knockdown of *LINC00857*. Cell viability was normalized to non‐cisplatin‐treated cells. Bars and graphs represent mean ± SD (*t* test, *P < .05; **P < .01; ***P < .001)

*LINC00857* upregulation was found to be associated with decreased platinum‐based chemotherapy response. Therefore, we evaluated whether knockdown of *LINC00857* could sensitize T24 and UM‐UC‐3 bladder cancer cells to cisplatin. Using *LINC00857*‐specific siRNAs, lncRNA levels were reduced more than 90%, compared to negative control transfected cells (Figure 3A). *LINC00857* knockdown sensitized both T24 and UM‐UC‐3 cells to cisplatin (Figures 3B,C). The dose of cisplatin needed to reduce cell viability by 50% (IC50 value) is more than 2‐fold lower in cells with knockdown of *LINC00857* compared to control cells. Interestingly, knockdown of *LINC00857* led to increased cisplatin sensitivity of UM‐UC‐3 cells in a wide range of cisplatin concentrations (Figure 3B). In contrast, the sensitivity of T24 cell line was found to be increased only in a narrow cisplatin dose range (Figure 3C).

### 3.4 *LINC00857* regulates expression of genes involved in chemoresistance

**Figure 4:**
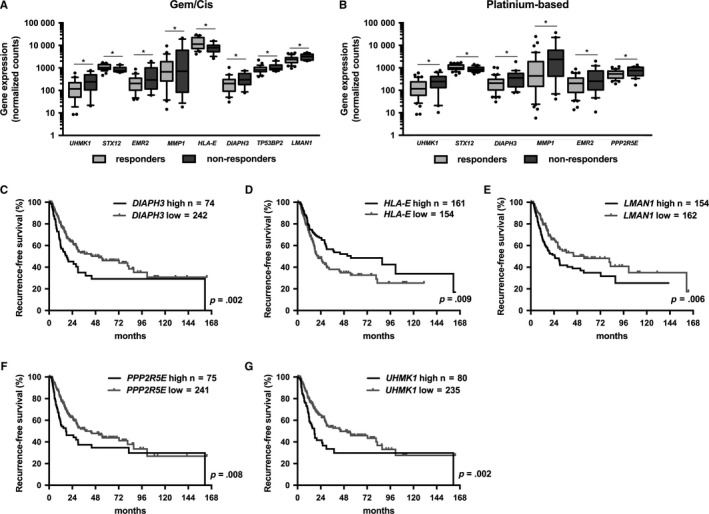
Expression of *LINC00857* target genes in MIBC. Association of *LINC00857* target gene expression with response to (A) gemcitabine/cisplatin (Gem/Cis; responders, n = 29; nonresponders, n = 12) and (B) platinum‐based chemotherapy (responders, n = 40; nonresponders n = 20). Kaplan‐Meier analysis showing correlation between recurrence‐free survival and (C) *DIAPH3*, (D) *HLA‐E*, (E) *LMAN1*, (F) *PPP2R5E*, and (G) *UHMK1* expression levels in the MIBC cohort of TCGA. The high and low *LINC00857* expression groups were determined based on the 75th and 25th percentiles. The survival curves were analyzed using the log‐rank test

To further evaluate how *LINC00857* contributes to platinum‐based chemotherapy response in bladder cancer, we evaluated the expression levels of *LINC00857* target genes that were identified in lung cancer.^20^ In MIBC, the expression of *DIAPH3*,* EMR2*,* MMP1*,* STX12*, and *UHMK1* was found to be significantly associated with response to Gem/Cis (Figure 4A) and other platinum‐based chemotherapy (Figure 4B). *PPP2R5E* expression was significantly upregulated in the non‐responders to platinum‐based chemotherapy (Figure 4B). Additionally, the expression of *HLA‐E*,* LMAN1*, and *TP53BP2* correlated significantly with response to Gem/Cis chemotherapy (Figure 4B). High expression of *DIAPH3*,* LMAN1*,* PPP2R5E*, and *UHMK1* and low *HLA‐E* expression were significantly associated with shorter recurrence‐free survival (*P* = .002, *P* = .006, *P* = .008, *P* = .002, and *P* = .009 resp.; Figure 4C‐G). Moreover, high *DIAPH3, HLA‐E*, and *LMAN1* expression was significantly associated with shorter overall survival (*P* = .043, *P* = 0.018, and *P* = .014, resp.; Figure S2), and high *PPP2R5E* and *UHMK1* expression showed a trend toward association with shorter overall survival in MIBC (*P* = .052 and *P* = .078, resp.; Figure S2).

**Figure 5:**
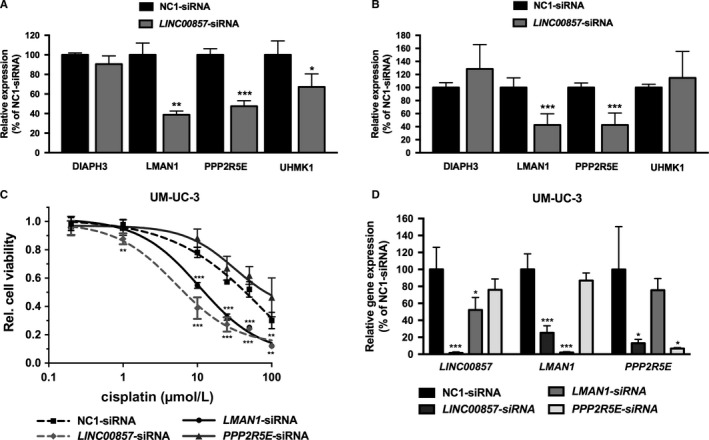
*LINC00857* target gene expression and cisplatin sensitization in T24 and UM‐UC‐3 bladder cancer cells. Expression of *LINC00857* target genes *DIAPH3*,*LMAN1*,*PPP2R5E*, and *UHMK1* in (A) T24 and (B) UM‐UC‐3 bladder cancer cell lines upon *LINC00857* knockdown. All data are compared to NC1‐siRNA‐transfected cells. C, Cisplatin sensitivity of UM‐UC‐3 cells 48 h after siRNA‐mediated knockdown of *LMAN1* and *PPP2R5E*. Cell viability was normalized to non‐cisplatin‐treated cells. D, The efficiency of gene knockdown in siRNA‐transfected UM‐UC‐3 cells. Gene expression levels were normalized against *GAPDH* housekeeping gene expression levels and then compared to negative control (NC1) siRNA‐transfected cells. Bars and graphs represent the mean ± SD (*t* test, *P < .05; **P < .01; ***P < .001)

We further evaluated the expression of *DIAPH3*,* LMAN1*,* PPP2R5E*, and *UHMK1* in bladder cancer cell lines upon *LINC00857* knockdown. The expression levels of *LMAN1* and *PPP2R5E* were found to be significantly downregulated when *LINC00857* expression was reduced in T24 and in UM‐UC‐3 cells (*P* < .01 and *P* < .001, resp.; Figure 5A,B). Knockdown of *LMAN1*, but not *PPP2R5E*, also sensitized UM‐UC‐3 cells to cisplatin to a similar degree as with *LINC00857* knockdown (Figure 5C). Gene knockdown was confirmed by semiquantitative RT‐PCR (Figure 5D).

## 4 DISCUSSION

Muscle‐invasive bladder cancer is a life‐threatening disease as half of the diagnosed patients show progression after initial treatment, develop metastasis, and died of the disease. The use of platinum‐based chemotherapy (either in the neoadjuvant setting or for metastatic patients) has been shown to significantly improve overall survival of the responding patients.^3^, ^4^ However, until now, no prognostic and predictive biomarkers, which allow for the selection of patients who will benefit from chemotherapy or monitoring of the efficacy of the treatment, are used in the clinical management of MIBC.^4^ Therefore, there is a need for new biomarkers as well as therapeutic targets in MIBC. Here, we analyzed the expression of lncRNAs that may be associated with the response to platinum‐based chemotherapy using MiTranscriptome lncRNA expression data in patients with MIBC.

In our study, high *LINC00857* expression was found to be associated with the lack of response to platinum‐based chemotherapy. Previously, *LINC00857* was identified as one of the most upregulated lncRNAs in lung cancer. *LINC00857* knockdown has been shown to reduce cell viability, migration, and invasion in vitro and in vivo by regulating a variety of genes involved in cell cycle progression. Furthermore, similarly to what we have found in bladder cancer, high *LINC00857* expression was associated with shorter overall survival of patients with lung cancer suggesting its oncogenic role in cancer.^20^

Recently, a panel of 5 lncRNAs including *LINC00857* was shown to accurately detect the presence of gastric cancer in blood. The high expression levels of these lncRNAs were also found to be correlated with tumor aggressiveness and lymphatic metastasis,^21^ an association we also found for *LINC00857* in MIBC. Moreover, lncRNA expression levels decreased postoperatively, suggesting a potential to monitor therapy efficacy.^21^ A liquid biopsy has been shown to facilitate evaluation of a variety of potential biomarkers including cell‐free DNA, CTCs, cell‐free proteins, peptides, exosomes, and circulating RNAs (mRNAs, microRNAs, and lncRNAs) and is currently being investigated in urological malignancies as well.^22^ The expression levels of 3 lncRNAs: *MEG3*,* SNHG16*, and *MALAT1* were recently shown to accurately detect bladder cancer in serum.^23^ Therefore, the usefulness of *LINC00857* and other prognostic lncRNAs as a noninvasive, blood‐based biomarker should be evaluated in blood collected from patients with MIBC.

In our study, *LINC00857* expression in the primary tumor was shown to predict MIBC progression and response to chemotherapy. The accurate selection of patients that will not respond to chemotherapy could lead to earlier selection of other treatment regimens, leading to improved overall survival of the chemotherapy nonresponding patients. Recently, atezolizumab (anti‐PDL1 antibody) and other checkpoint inhibitors were approved for the treatment of patients with advanced bladder cancer, who progressed after platinum‐based chemotherapy, and numerous clinical trials evaluating their clinical effectiveness are ongoing.^24^

Several methods have been developed to target lncRNA expression, including antisense oligonucleotides, siRNAs, and (deoxy)ribozymes, representing a possibility of therapeutic intervention.^25^ For example, targeting *SAMMSON* (a lncRNA upregulated in melanoma) in vivo by intravenous treatment with antisense oligonucleotides has been shown to significantly suppress tumor growth in patient‐derived xenograft models.^26^ *LINC00857* expression levels were found to be upregulated in the nonresponders group in MIBC, therefore targeting *LINC00857* could potentially sensitize bladder cancer cells to chemotherapy.

Multiple *LINC00857* target genes were identified in the lung cancer study.^20^ In our study, expression levels of *DIAPH3*,* EMR2, MMP1*,* PPP2R5E, STX12*, and *UHMK1* were shown to be associated with platinum‐based chemotherapy response. Moreover, high expression of *DIAPH3, LMAN1, PPP2R5E*, and *UHMK1* was correlated with shorter progression‐free and/or overall survival. Knockdown of *LINC00857* in bladder cancer cell lines resulted in decreased expression of *LMAN1* and *PPP2R5E*, and loss of *LMAN1* expression sensitized cells to cisplatin to a similar degree as knockdown of *LINC00857*. *LMAN1* (a mannose‐specific lectin) is responsible for transport of glycosylated proteins from endoplasmic reticulum to Golgi.^27^ It was shown to be frequently mutated in microsatellite instability‐positive gastric^28^ and colorectal cancer.^27^ Moreover, lack of LMAN1 expression influenced transport of antiangiogenic and growth inhibiting protein‐ A1AT, contributing to colorectal carcinogenesis.^27^ *PPP2R5E,* a regulatory subunit of protein phosphatase 2A (*PP2A*) was found to be downregulated in colorectal cancer^29^ and leukemia.^30^ Suppression of *PPP2R5E* sensitizes tumor cells to DNA damaging agents through regulation of cell cycle and DNA repair pathways.^31^ *PPP2R5E* has also been found to have anti‐ and proapoptotic properties and to be a substrate for caspase‐3 during cisplatin‐induced apoptosis.^32^ As *LINC00857* was found to be enriched in the cytoplasmic fraction,^20^ it is likely that *LINC00857* regulates the expression of *LMAN1* and *PPP2R5E* and other target genes by affecting mRNA stability or promoting its translation leading to resistance to platinum‐induced apoptosis.

One of the limitations of our study is studying a single lncRNA as potential prognostic and predictive biomarker in MIBC. Large‐scale initiatives, like the TCGA consortium, have identified several MIBC subtypes characterized by distinct genetic and transcriptional alterations and thus clinical outcomes. The lncRNAs expression was shown to be concordant with the mRNA‐defined subtypes but also further discriminated subgroups associated with survival.^33^, ^34^ This shows that MIBC is highly a heterogeneous disease; therefore, likely a combination of multiple markers predicting progression and/or therapy response is needed to accurately stratify patients. We showed that *LINC00857* expression is associated with response to platinum‐based chemotherapy; however, the number of used patients is low. The studies on lncRNAs expression as potential predictive biomarkers for platinum‐based chemotherapy in MIBC are still limited and focused on protein‐coding genes. Moreover, MIBC patients’ cohorts with comprehensive lncRNAs sequencing and clinical data are lacking. More studies in larger platinum‐treated patient cohorts are needed to validate *LINC00857* and other lncRNAs as markers of chemotherapy response in bladder and other cancer types (also in a neoadjuvant setting).

In conclusion, our data indicate that lncRNAs play an important role in MIBC progression and response to platinum‐based chemotherapy. Further studies should focus on how *LINC00857* and other lncRNAs regulate pathways underlying MIBC aggressiveness and chemotherapy resistance. This may ultimately lead to the development of new prognostic and predictive biomarkers and possibly new treatment options for MIBC.

## CONFLICT OF INTEREST

The authors declare no conflict of interest.
