# Supplementary material for: LINC00857 expression predicts and mediates the response to platinum‐based chemotherapy in muscle‐invasive bladder cancer
Source: Cancer Med. 2018 Jun 1;7(7):3342–50. doi: 10.1002/cam4.1570 (PMC6051137; doi:10.1002/cam4.1570)

**Supplementary Table 1.** Characteristics of platinum-based chemotherapy-treated patients from TCGA cohort used in MiTranscriptome lncRNAs analysis and *LINC00857*-target genes analysis ^20^.

| **Parameter** | **MiTranscriptome** | | **TCGA** | |
| --- | --- | --- | --- | --- |
|  | **Responsive**  **n=7^*^** | **Progressive**  **n=10** | **Responsive**  **n=40^**^** | **Progressive**  **n=20** |
| **Age at diagnosis**  (average, range) | 62.6  (50-72) | 64.6  (54-68) | 64.8  (45-79) | 64.7  (54-75) |
| **Gender** |  |  |  |  |
| female | 3 | 3 | 13 | 7 |
| male | 4 | 7 | 27 | 13 |
| **Stage** |  |  |  |  |
| T2 | 2 | 5 | 10 | 8 |
| T3 | 4 | 5 | 21 | 9 |
| T4 | 1 | 0 | 8 | 3 |
| **Chemotherapy type** |  |  |  |  |
| cisplatin/gemcitabine | 4 | 7 | 29 | 12 |
| cisplatin/gemcitabine/taxol | 0 | 0 | 1 | 0 |
| cisplatin/gemcitabine/paclitaxel | 0 | 0 | 1 | 0 |
| cisplatin/adriamycin/vinblastine/methotrexate | 2 | 0 | 2 | 0 |
| cisplatin/doxorubicin/vinblastine/methotrexate | 0 | 1 | 2 | 2 |
| carboplatin/gemcitabine | 0 | 1 | 3 | 4 |
| carboplatin/etoposide | 1 | 0 | 1 | 0 |
| carboplatin/etoposide/doxorubicin | 0 | 0 | 1 | 0 |
| carboplatin/5-FU | 0 | 0 | 0 | 1 |
| carboplatin/taxol | 0 | 1 | 0 | 1 |

^*^, partial response (n=1), stable disease (n=1);**, partial response (n=5), stable disease (n=2).

**Supplementary Table 2.** Primers and siRNA sequences.

| Primers sequences | | |
| --- | --- | --- |
| Gene | **Forward (5`🡪3`)** | **Reverse (5`🡪3`)** |
| *LINC00857* | CGTCTTCCCCTCCATCGTG | GATGGGGTACTTCAGGGTGA |
| *HP1BP3* | TGGAATATGCAATCTTGTCTGC | GAACCCTTTCCCAGAGATCTG |
| *UHMK1* | TACAGTCAGATCTCAGGAATGGA | CGGCATTCACCACTGCTTTAC |
| *DIAPH3* | GCGGTATGCATTGTAGGGGA | CCGGAGGCCTTCCACAATAC |
| *PPP2R5E* | AGATCCTTCACTCACAGAACC | AGTTCCCCAAGGAACATGAC |
| *TP53BP2* | GCCAAGGCTCTGCTTCTGTA | ACATTGAGAACGGACGCACT |
| siRNAs sequences | | |
| Name | **Sense (5`🡪3`)** | **Antisense (5`🡪3`)** |
| *LINC00857* siRNA ^20^ | GGCUAUGUGCUGUGAACAAUU | UUGUUCACAGCACAUAGCCUU |
| *LMAN1* siRNA | GCAGAUUACUCAACAAGAATT | (Ambion AM16708, s8219) |
| *PPP2R5E* siRNA | GGAGCUAUUUGACAGCGAATT | (Ambion AM16708, s10994) |
| NC1 siRNA | Silencer Negative Control No.1 | (Ambion, AM4611) |
|  |  |  |

**Supplementary Table 3.** The most de-regulated lncRNAs associated with platinum-based chemotherapy response in MIBC.

| Transcript^#^ | Name | Gene | Av  (CR/PR/SD) | Av  (PD) | P-value  (t-test) |
| --- | --- | --- | --- | --- | --- |
| T115838 | ***CAT1735*** | Overlaps with *RP11-109D20.2,* antisense to *SORD* | 0.451 | 1.177 | 0.002 |
| T032228 | ***BRCAT81*** | *RP5-1139B12.2,* antisense to *OBSCN* | 0.032 | 0.191 | 0.003 |
| T324413 | ***KCCAT484*** | Antisense to *EGFR* | 0.028 | 0.187 | 0.005 |
| T100139 | ***CAT1637*** | *AL589743.1* | 2.066 | 0.512 | 0.006 |
| T285343 | ***LVCAT3.3*** | Novel | 0.302 | 0.006 | 0.007 |
| T081667 | ***CAT1544*** | Novel | 0.237 | 0.021 | 0.008 |
| T049520 | ***CAT1309.1*** | Antisense to *TIAL1* | 2.149 | 0.818 | 0.008 |
| T255255 | ***CAT475.2*** | *KCNMB2-AS1* | 0.138 | 0 | 0.008 |
| T187946 | ***THCAT573*** | Overlaps with *AC011247.3,* antisense to *HNRNPLL* | 0.136 | 0.057 | 0.008 |
| T248567 | ***MBAT60*** | Antisense to *ZBTB20* | 0.149 | 0.040 | 0.010 |
| T287339 | ***EPB41L4A-AS1*** | *EPB41L4A-AS1* | 11.04 | 4.685 | 0.010 |
| T083363 | ***LSCAT235*** | Novel | 0.115 | 0.024 | 0.010 |
| T285341 | ***LVCAT3.1*** | Overlaps with *NBPF22P* and *CTC-261N6* | 0.288 | 0.024 | 0.011 |
| T138315 | ***AMAT2*** | Overlaps with *RP5-114A6*, antisense to *PIEZO1* | 0.118 | 0.340 | 0.011 |
| T045001 | ***LINC00857*** | *LINC00857* | 0.116 | 0.399 | 0.011 |
| T108430 | ***CAT1695*** | Novel | 0.233 | 0.030 | 0.013 |
| T192672 | ***CAT266.1*** | Novel | 0.728 | 0.185 | 0.013 |
| T180224 | ***KCCAT467*** | Antisense to *ZNF83* | 0.316 | 0.076 | 0.013 |
| T171341 | ***THCAT254*** | Overlaps with *CTC-513N18.6*, antisense to *ZNF826P* | 0.286 | 0.043 | 0.014 |
| T203798 | ***TTN-AS1.5*** | *TIN-AS1* | 0.159 | 0.036 | 0.015 |
| T049521 | ***UTAT56*** | Antisense to *TIAL1* | 0.943 | 0.216 | 0.015 |
| T365807 | ***MBAT108*** | Novel | 2.263 | 1.473 | 0.016 |
| T246531 | ***CAT428*** | Overlaps with *RP11-389G6.5*, antisense to *ARL6* | 1.528 | 0.592 | 0.018 |
| T244703 | ***MEAT114*** | Antisense to *MAGI1* | 1.248 | 0.115 | 0.018 |
| T265271 | ***-*** | Novel | 0.356 | 0.106 | 0.018 |
| T004822 | ***LACAT2.2*** | *RP-340N1.2* | 0.046 | 0.368 | 0.019 |
| T093098 | ***THCAT124*** | Antisense to *POSTN* | 0.118 | 0.041 | 0.019 |
| T127668 | ***SMAT19*** | *MIR193BHG* | 0.054 | 0.282 | 0.019 |

#, MiTranscriptome identifier; Av, average expression (FPKM); CR, complete response; PR, partial response; SD, stable disease; PD; progressive disease. LncRNAs with average expression lower than 0.1 FPKM in both groups (responders and non-responders) were excluded.

**Supplementary Table 4.** The correlation between clinico-pathological characteristics and *LINC00857* expression levels.

| Characteristic | No.^*^ |  | *LINC00857* expression^#^ |  |
| --- | --- | --- | --- | --- |
|  |  | **Low**  **No. (%)** | **High**  **No. (%)** | **P-value^$^** |
| Age (years) |  |  |  |  |
| ≤60 | 38 | 29 (76%) | 9 (24%) | 0.669 |
| >60 | 84 | 61 (73%) | 23 (27%) |  |
| Sex |  |  |  |  |
| Female | 34 | 25 (74%) | 9 (26%) | 0.970 |
| Male | 88 | 65 (74%) | 23 (26%) |  |
| Stage |  |  |  |  |
| T2 | 42 | 29 (69%) | 13 (31%) | 1 |
| T3 | 62 | 48 (77%) | 14 (23%) | 0.342 |
| T4 | 17 | 13 (76%) | 4 (24%) | 0.572 |
| Grade |  |  |  |  |
| low | 5 | 5 (83%) | 1 (17%) | 0.608 |
| high | 115 | 85 (74%) | 30 (26%) |  |
| Subtype |  |  |  |  |
| Papillary | 33 | 25 (76%) | 8 (24%) | 0.881 |
| Non-papillary | 86 | 64 (74%) | 22 (26%) |  |
| Lymph nodes |  |  |  |  |
| Positive (N1-3) | 40 | 23 (58%) | 17 (42%) | 0.003 |
| Negative (N0) | 72 | 60 (83%) | 12 (17%) |  |
| Progression |  |  |  |  |
| Disease-free | 41 | 37 (90%) | 4 (10%) | 0.019 |
| Progressed | 50 | 35 (70%) | 15 (30%) |  |

^*^, not all data available for all patients; ^#^, divided into high (>75^th^ percentile) and low (<75^th^ percentile);

^$^, Chi-squared test.

**Supplementary Table 5.** Univariate and multivariate analysis of *LINC00857* expression levels as prognostic factor for MIBC overall survival.

| **Factor** | **Univariate** | | | **Multivariate** | | |
| --- | --- | --- | --- | --- | --- | --- |
|  | **Exp (B)** | **P value** | **95% CI** | **Exp (B)** | **P value** | **95% CI** |
| **Stage** | 2.08 | 0.001 | 1.335-3.265 | 1.854 | 0.010 | 1.156-2.975 |
| **Lymph node status** | 2.073 | 0.006 | 1.226-3.506 | 1.431 | 0.239 | 0.788-2.598 |
| **Age** | 1.512 | 0.176 | 0.830-2.753 | 1.388 | 0.349 | 0.699-2.758 |
| **Gender** | 0.899 | 0.712 | 0.513-1.578 | 0.704 | 0.891 | 0.491-1.616 |
| ***LINC00857* expression^#^** | 2.187 | 0.004 | 1.286-3.719 | 2.058 | 0.025 | 1.097-3.860 |

Exp (B), B coefficient representing odds ratio; CI, confidence intervals; ^#^, divided into high (>75^th^ percentile) and low (<75^th^ percentile).

**Supplementary Table 6.** Univariate and multivariate analysis of *LINC00857* expression levels as prognostic factor for MIBC recurrence-free survival.

| **Factor** | **Univariate** | | | **Multivariate** | | |
| --- | --- | --- | --- | --- | --- | --- |
|  | **Exp (B)** | **P value** | **95% CI** | **Exp (B)** | **P value** | **95% CI** |
| **Stage** | 1.788 | 0.019 | 1.102-2.901 | 1.749 | 0.038 | 1.031-2.967 |
| **Lymph node status** | 2.130 | 0.013 | 1.170-3.878 | 1.794 | 0.086 | 0.920-3.500 |
| **Age** | 1.414 | 0.263 | 0.771-2.595 | 1.347 | 0.442 | 0.631-2.875 |
| **Gender** | 0.962 | 0.900 | 0.525-1.762 | 0.881 | 0.727 | 0.432-1.796 |
| ***LINC00857* expression^#^** | 2.000 | 0.027 | 1.082-3.695 | 1.509 | 0.317 | 0.674-3.379 |

Exp (B), B coefficient representing odds ratio; CI, confidence intervals; ^#^, divided into high (>75^th^ percentile) and low (<75^th^ percentile).

**Supplementary Figure 1.** Kaplan-Meier analysis of *LINC00857* expression levels with (**A**) recurrence-free survival (high n=43; low n=48) and (**B**) overall survival (high n=60; low n=60) of MIBC patients in the MiTranscriptome cohort. The patients are divided into the high and low expression groups based on the median gene expression in all MIBC cases. The survival curves were analyzed using the Log-rank test.


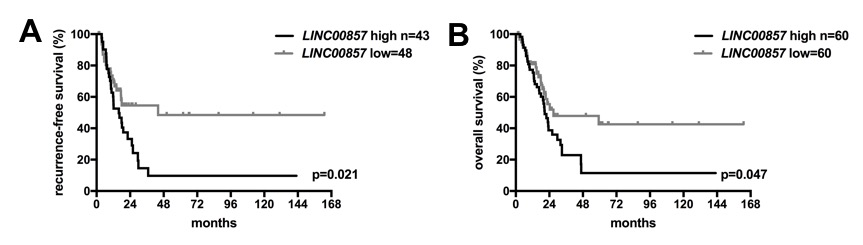


**Supplementary Figure 2.** Kaplan-Meier analysis of *LINC00857* target genes expression levels, (**A**) *DIAPH3*, (**B**) *HLA-E*, (**C**) *LMAN1,* (**D**) *PPP2R5E* and (**E**) *UHMK1*, with overall survival of MIBC patients in the TCGA cohort (33). The patients are divided into the high and low expression groups based on the 75^th^ and 25^th^ percentiles (>75^th^ percentile, high; <25^th^ percentile, low) for *DIAPH3*, *PPP2R5E* and *UHMK1* and based on median expression for *HLA-E* and *LMAN1*. The survival curves were analyzed using the Log-rank test.


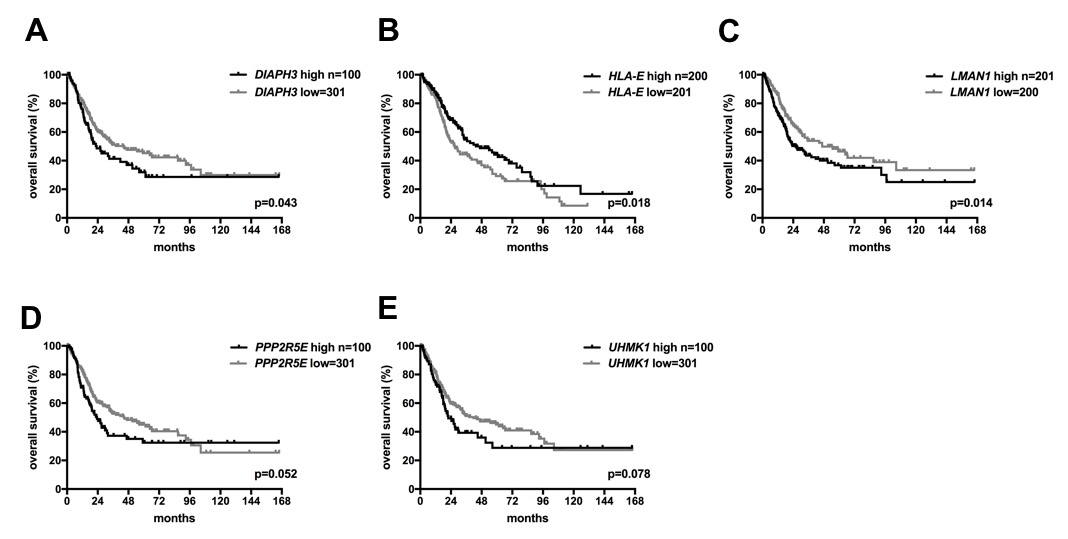

Supplement: Supplementary file 1 [file CAM4-7-3342-s001.docx]
